# Supplementary material for: Estrogen profiling in blood and brain: Effects of season and an aggressive interaction in a songbird
Source: PLoS One. 2025 Dec 15;20(12):e0326727. doi: 10.1371/journal.pone.0326727 (PMC12704853; doi:10.1371/journal.pone.0326727)
Supplement: Attachment — Submitted filename: Response to Reviewers.docx [file pone.0326727.s002.docx]

**Manuscript ID: PONE-D-25-30431**

**We sincerely thank the editor and reviewers for their thoughtful evaluation of our manuscript. We are encouraged by their positive feedback, noting that the study is well written and clearly presented, with extensive validations, well-documented results, and strong methodological development for measuring multiple estrogens. We also appreciate their constructive suggestions, which have helped us further improve the clarity and rigor of the work. We have addressed every comment point by point, as detailed below (changes in the revised manuscript are highlighted in blue). We believe that the revisions have substantially strengthened the manuscript.**

Journal Requirements:

1. Please ensure that your manuscript meets PLOS ONE's style requirements, including those for file naming. The PLOS ONE style templates can be found at

**Response: We have revised the manuscript and all associated files to ensure compliance with PLOS One’s style requirements, including file naming.**

2. To comply with PLOS One submissions requirements, in your Methods section, please provide additional information regarding the experiments involving birds and ensure you have included details on (1) methods of sacrifice, and (2) efforts to alleviate suffering.

**Response: We have revised the Methods section to include the requested details. Specifically, we now describe the method of sacrifice used, as well as the measures taken to minimize stress and alleviate suffering during handling and experimental procedures.**

3. In your Methods section, please provide additional information regarding the permits you obtained for the work. Please ensure you have included the full name of the authority that approved the field site access and, if no permits were required, a brief statement explaining why.

**Response: Animal use was approved by the University of British Columbia Animal Care Committee (Protocol #A19-0074), and fieldwork was conducted under a permit issued by the Canadian Wildlife Service (Permit #SC-BC-2019-0012). We have included the required information in the Methods section (lines 119-120).**

4. Thank you for stating the following financial disclosure:

“Agencia Nacional de Investigación e Innovación (POS_EXT_2016_1_134441) to CJ

Zoology Graduate Fellowship, University of British Columbia (6444) to CJ

Canadian Graduate Scholarships-Master's, Canadian Institutes of Health Research (6556) to MQL

Faculty of Medicine Graduate Award, University of British Columbia (6442) to MQL

Canadian Institutes of Health Research Operating Grant (169203) to KKS

Canada Foundation for Innovation Grant (32631) to KKS”

Please state what role the funders took in the study. If the funders had no role, please state: 'The funders had no role in study design, data collection and analysis, decision to publish, or preparation of the manuscript.'

If this statement is not correct you must amend it as needed.

Please include this amended Role of Funder statement in your cover letter; we will change the online submission form on your behalf.

**Response: We confirm that the funders had no role in study design, data collection and analysis, decision to publish, or preparation of the manuscript. We have added this statement to the cover letter as requested.**

5. We note that you have included the phrase “data not shown” in your manuscript. Unfortunately, this does not meet our data sharing requirements. PLOS does not permit references to inaccessible data. We require that authors provide all relevant data within the paper, Supporting Information files, or in an acceptable, public repository. Please add a citation to support this phrase or upload the data that corresponds with these findings to a stable repository (such as Figshare or Dryad) and provide and URLs, DOIs, or accession numbers that may be used to access these data. Or, if the data are not a core part of the research being presented in your study, we ask that you remove the phrase that refers to these data.

**Response: We have removed the phrase “data not shown” from the manuscript.**

6. Please include your full ethics statement in the ‘Methods’ section of your manuscript file. In your statement, please include the full name of the IRB or ethics committee who approved or waived your study, as well as whether or not you obtained informed written or verbal consent. If consent was waived for your study, please include this information in your statement as well.

**Response: We thank the editor for this reminder. We have revised the Methods section to include the full ethics statement, specifying the protocol approved by the UBC Animal Care Committee (line 120). The study did not include human participants, and informed consent was not applicable.**

7. If the reviewer comments include a recommendation to cite specific previously published works, please review and evaluate these publications to determine whether they are relevant and should be cited. There is no requirement to cite these works unless the editor has indicated otherwise.

**Response: not applicable**

Reviewer #1:

This paper describes a new highly sensitive method to measure multiple estrogen by a LC/MS_MS. The method appears to be quantitative and precise. It was applied to the song sparrow, a model well-known in the field of Behavioral Neuroendocrinology. In general the paper is very well written and extremely clear. I have no general complaint about the manuscript. Just a few comments about specific items.

I cannot comment in a critical manner about the assay method even if I can understand it but I have no personal experience with the different options. This being said, the results are very well documented. Extensive validations have been performed and confirm the accuracy and specificity of the results.

**Response: We are very pleased that the reviewer found the results to be well documented and the validations to confirm the accuracy and specificity of our method. We appreciate this encouraging feedback regarding the rigor and reliability of our approach.**

It is said on line 107 that all birds were caught by mist nets and song playback but then on line 218-222, it is said that some birds were caught by STI and other without decoy and loudspeaker./playback. Then a bit later the CON and STI are exposed to playback? How are they different then: Only by the presence/absence of decoy.? Please clarify.

**Response: We thank the reviewer for this helpful request for clarification. We have revised the Methods section to clearly distinguish between experimental treatments and the capture procedure. All birds, including those used for method development and those assigned to the experimental groups, were captured in the same way—using a mist net and brief conspecific playback to attract an individual to the net. Playback was necessary for capture, as birds are otherwise unlikely to approach the net.**

**For the experimental treatment, subjects were randomly assigned to one of two groups: “STI,” in which birds were exposed to conspecific playback (from a speaker) and a live decoy in a cage for 10 min, or “CON,” in which birds were exposed to a silent speaker and an empty cage for 10 min.**

**Next, subjects from both groups were captured with a mist net, and a brief amount of conspecific playback (maximum of 5 min; breeding CON: 2.2 ± 0.6 min, breeding STI: 1.4 ± 0.6 min, non-breeding CON: 2.0 ± 0.4 min, non-breeding STI: 1.5 ± 0.4 min) was used to lure subjects into the net. The 5 min maximum was designed to limit exposure of subjects to playback at capture. Birds that did not enter the net within 5 min were excluded from the study. These details have now been clarified in the text (lines 236-240).**

It is unfortunate that estrogens concentrations are expressed in ng/g in figure 3 and in nM in figure 4. This should be standardized one way or the other. If you select the units used in figure 3, I would suggest to change to pg/g since this would remove unnecessary zeros.

**Response: We thank the reviewer for this comment regarding units. We deliberately chose to present concentrations in ng/g in Figure 3 and in nM in Figure 4 because these figures serve different purposes. Presenting values in ng/g shows absolute levels in specific brain regions and blood, while presenting values in nM allows for direct comparison of relationships between estrogens that differ in molecular weight. We believe that this dual presentation provides complementary perspectives that are useful for interpreting the data. However, if the editor prefers a uniform unit throughout, we are happy to adjust accordingly. We have now clarified this point in the respective figure legends.**

One key observation here is that, contrary to expectations, no change in estrogens was observed after 10 min of aggressive interaction while previous studies had observed an increase in testosterone and androstenedione after an STI (line 455), and an increase in E2 had been detected after STI in white-crowned sparrow (line 469). This discrepancy is adequately discussed but It would be important to remind the reader what was the latency in the previous studies compared to this one (see line 462).

**Response: We have clarified the time frames in each study (lines 488-495).**

It is also puzzling that all estrogens were undetectable during the non breeding season contrary to what could have been expected based on previous studies of this lab. showing central production of sex steroids from adrenal DHEA. This should also be discussed.

**Response: The ability of the brain to locally synthesize sex steroids from inactive precursors, such as dehydroepiandrosterone (DHEA), has been proposed as a mechanism to support non-breeding aggression. Earlier studies from our laboratory reported circulating DHEA levels based on radioimmunoassays. However, when a more specific method (mass spectrometry) was applied, circulating DHEA levels were much lower or non-detectable. We therefore speculate that other precursors, such as DHEA fatty acid esters or progesterone (which we detected in blood and brain during the non-breeding season (Jalabert et al., 2021)), could serve as substrates for local androgen and estrogen synthesis. It is important to note that the present method was specifically optimized for the quantification of multiple estrogens, and other steroid classes (e.g., androgens, progestogens) were not measured. This has now been clarified in the text (lines 470-476).**

Reviewer #2:

The manuscript entitled “Estrogen profiling in blood and brain: effects of season and an aggressive interaction in a songbird" by Jalabert and colleagues established a new protocol for the development of brain estrogens. Subsequently they also attempted to compare brain estrogen levels between breeding and non-breeding season. Topic is more relevant to protocol based journals and biological functions are weakly addressed. My specific comments are:

1. Abstract lacks clarity specifically methodology related with animal experimentation though detailed methodology for establishing new protocol has been provided

**Response: The Abstract has been revised to be clearer about the animal experimentation.**

2. Line 104-15: It does not clear how many birds were procured at each season.

**Response: In total, we used 45 individuals: 5 for method validation and 40 for the behavioral experiment (10 per group per season). We have clarified this information in the Methods section (lines 110-111).**

3. During breeding season samplings were executed for 16 days while non breeding season for a month time (almost double time). What is the rationale behind it? Further, what was the gonadal/reproductive status of the birds procured during the breeding phase? As individual

gonadal status may vary significantly and hence alter the reproduction linked behaviour.

**Response: Our aim was to complete sampling in as short a time as possible within each season, in order to minimize variability. In the breeding season, we were able to reach the target sample size within 16 days. In the non-breeding season, weather (rain, snow, etc.) did not allow us to conduct field work as often, which extended the sampling period. We worked in the middle on the non-breeding season, to ensure that all subjects were clearly not in breeding condition.**

**Regarding reproductive status, all breeding males showed an enlarged cloacal protuberance and large testes, typical of reproductively active males in this species. In the non-breeding season, all birds sampled had a small cloacal protuberance and fully regressed testes, and were past molt. These clarifications have been added to the Methods section (lines 107-109).**

4. What is the natural breeding time in the wild population?

**Response: For the song sparrow population in the** **Pacific Northwest, breeding typically occurs from March through August. We have added this information and the respective citation in the text (lines 61-62).**

5. Please provide protocol number.

**Response: We have now included the protocol number in the Methods section (line 120).**

6. How individual behaviour of wild bird has been precisely monitored without mixing with

two or more individuals (line 221 to 224).

**Response: Song sparrows are strongly territorial, with well-defined and non-overlapping territories. We first located a focal individual and identified its territory. Because of this territorial behavior, when we conducted the simulated territorial intrusion (STI) within a focal male’s territory, only that individual responded to and closely approached the stimulus. This ensured that behavioral observations and subsequent sampling were restricted to a single subject. We have clarified this point in the Methods section (lines 233-235).**

7. Line 240, please provide details of make and model of cryostat used for sectioning.

**Response: We have added this information in the Methods section (line 253).**

8. Statistical analysis section line 256-257 mentioned using one way ANOVA while result

section line 347 to 349 talks about main effect and interaction. These two are contradictory.

**Response: We thank the reviewer for catching this inconsistency. The correct analysis was a two-way ANOVA, as described in the Results section. We have corrected the text in the Statistical Analysis section accordingly (lines 269-270).**

9. How individual songs were recorded for wild bird? I don’t see data related to it.

**Response: We did not make audio recordings of the focal birds. Instead, the vocal responses of each focal male during the STI were monitored directly in the field and documented in real time in field notebooks. This approach allowed us to quantify the number of songs and other behaviors during behavioral trials. We have clarified this in the Methods section (lines 230-231).**

10. Results lacks clarity and graphical resolution and representation is poor. It lacks

necessary information.

**Response: If the reviewer could provide specific comments on which aspects of the Results section or figures lack clarity, we would be happy to address them directly. Regarding figure quality, all images were prepared and uploaded at the resolution and format required by PLOS One guidelines.**

11. Despite robust aggressive responses to the STI, estrogen levels did not differ between

STI and control subjects in either season. Could it be because of sensitivity of the assay?

**Response: While we cannot fully exclude this possibility, we consider it unlikely that the lack of differences in estrogens between STI and control groups was due to assay sensitivity. The assay we developed has very low lower limits of quantification (0.02 pg for E_1_ and 0.05 pg for 17β-E_2_) and is among the most sensitive methods for estrogens. Moreover, we were able to detect regional and seasonal differences in estrogen levels with this method.**

**Other explanations seem more likely. First, the timeframe we used for sampling may not have captured transient changes in estrogens. Second, the relevant analyte(s) might not have been quantified here. Third, sample processing could affect measured estrogen levels, as oxidation and degradation may occur during freeze-thaw cycles (Kawato et al., 2025). Therefore, sample processing might contribute to the lack of treatment differences – but we still detect regional and seasonal differences. This information and the citation have been added to the manuscript (lines 500-502).**
